# Immunohistochemical localization of galectin-3 in the pig retina during postnatal development

**Published:** 2009-09-26

**Authors:** Jihoon Kim, Changjong Moon, Meejung Ahn, Hong-Gu Joo, Jae-Kwang Jin, Taekyun Shin

**Affiliations:** 1 Department of Veterinary Anatomy, College of Veterinary Medicine, Jeju National University, Jeju, South Korea; 2 Veterinary Medical Research Institute, Jeju National University, Jeju, South Korea; 3 Department of Veterinary Anatomy, College of Veterinary Medicine and Animal Medical Institute, Chonnam National University, Gwangju, South Korea; 4 Ilsong Institute of Life Science, Hallym University, Anyang, South Korea

## Abstract

**Purpose:**

The differential level and localization of galectin-3 protein were examined in the retinas of two-day-old pigs and six-month-old pigs.

**Methods:**

The retinas sampled from two-day-old and six-month-old pigs were analyzed by western blot and immunohistochemistry.

**Results:**

western blot analysis detected galectin-3 in both age groups, although the levels were significantly higher in six-month-old pigs. Immunohistochemical staining showed that galectin-3 was localized in the retinas of both two-day-old pigs and six-month-old pigs; the galectin-3 immunostaining was more intense in the six-month-old pig retina, as shown in the western blot analysis. Galectin-3 was expressed in glial cells, particularly in glutamine synthetase-positive Müller cells and their processes, across all retina layers in both age groups; however, it was not found in ganglion cells of the ganglion cell layer or neuronal cells of the inner and outer nuclear cell layers in either age group.

**Conclusions:**

This is the first demonstration that galectin-3 is detected in the retinas of two-day-old pigs and that the expression in Müller cells increases with postnatal development.

## Introduction

Galectin-3, which has a carbohydrate-recognition domain and an N-terminal domain rich in proline and glycine, is characterized by its unique structure among vertebrate galectins [1]. Fifteen galectins have been identified, and each contains a conserved carbohydrate-recognition domain of about 130 amino acids [2]. Galectin-3 in the cytoplasm of cells plays critical roles in regulating apoptosis [3], cell proliferation, differentiation, and survival [4,5]. Extracellular galectin-3 mediates the adhesion of leukocytes to the endothelium during inflammation [6]. Furthermore, galectin-3 is involved in mRNA splicing [4], metastasis [7], and angiogenesis [8]. Previously, we found galectin-3 in various mouse tissues in differing amounts [9] and in pig tissues, including testis and epididymis [10]. However, to date, galectin-3 expression has been rarely reported in the central nervous system (CNS) under normal conditions, whereas low expression is observed under pathological conditions, such as in macrophages/microglia during spinal cord demyelination [11], Müller cells during retinal degeneration [12], and in neoplastic astrocytes [13].

The retina is remote CNS tissue and is a model for studying CNS tissue development. The pig eye has been suggested as a novel model for human eye disease because the pig retina shares many similarities with the human retina [14-17]. The histological characteristics of the developing retina have been examined in fetal pigs [18]. In pigs, the number of retinal ganglion cells decreases with age [15], presumably due to oxidative stress [19]. Previously, it was suggested that galectin-3 increases in Müller cells of a degenerative rat retina, probably through endogenous anti-apoptosis [12].

The present study examined the expression and cellular localization of galectin-3 in the retinas of two-day-old and six-month-old pigs to evaluate the differential level and localization of galectin-3 during postnatal development in the pig retinas.

## Methods

### Animals and tissue samples

The eyes of six-month-old young adult pigs (n=5) were obtained immediately after butchering in a local slaughterhouse. Two-day-old pigs were obtained from a local farm (n=3). These were killed by CO_2_ inhalation. The anterior segment of the eyeball was removed, and the retinas were carefully dissected on ice. All the experiments were performed in accordance with the Guide for the Care and Use of Laboratory Animals in Jeju National University, Jeju, Korea.

For western blot analysis, retinal tissues were detached from the eyecups and kept in a deep freezer. For histology, the anterior segments of the eyes were dissected. The eyecups without anterior segments were fixed by immersion in 10% neutral-buffered formalin in phosphate-buffered saline (PBS; 137mM NaCl, 2.7mM KCl, 4.3mM Na_2_PO_4_, 1.4mM KH_2_PO_4_, pH 7.4) for 24 h. Chemicals for PBS were obtained from JUNSEI (Tokyo, Japan). Fixed tissues were dehydrated through a graded series of ethanol and embedded in tissue embedding medium (Paraplast®, Tyco healthcare group Lp, MA). The paraffin-embedded retinal tissues were cut 5μm on a rotary microtome (Leica, Nussloch, Germany).

### Antibodies

Approximately 1 mg/ml rat anti-galectin-3 monoclonal antibody was purified by affinity chromatography from the supernatants of hybridoma cells (clone TIB-166^™^, M3/38.1.2.8. HL.2; American Type Culture Collection, Manassas, VA). Mouse monoclonal anti-glial fibrillary acidic protein (GFAP; G-3893; Sigma-Aldrich, St. Louis, MO) was used to detect astrocytes while anti-glutamine synthetase (MAB302; Chemicon International, Temecula, CA) was employed to detect Müller cells [20]. A mouse monoclonal anti-β-actin antibody (A5441; Sigma-Aldrich) was used to detect β-actin labeling on western blots.

### Western blotting

Western blot analysis of the pig retina was performed as reported previously, with minor modifications [21,22]. In brief, retinal tissues were thawed in lysis buffer that consisted of 20 mM HEPES (pH 7.2; Sigma-Aldhrich, St. Louis. MO), 1% Triton X-100 (Amresco, Solon, OH), 1% deoxycholate (Sigma-Aldhrich), 0.1% sodium dodecyl sulfate (SDS; Bio-rad, Hercules, CA), 150 mM NaCl (Junsei, Tokyo, Japan), 10 μg/ml leupeptin (Sigma-Aldhrich), 10 μg/ml aprotinin (Sigma-Aldhrich), and 1 mM phenylmethylsulfonyl fluoride (Sigma-Aldhrich). Tissues were given 10 strokes in a Dounce homogenizer before they were sonicated for 10 s. The homogenates were centrifuged at 4 °C and 19,340× *g* for 20 min, and the lysate supernatant was obtained. Next, 40 µg of the lysate were dissolved in the SDS sample buffer and boiled. The homogenized tissue samples were electrophoresed under denaturing conditions in 10% SDS-polyacrylamide gels. Then, the proteins were electrotransferred in transfer buffer to a nitrocellulose transfer membrane (Schleicher and Schuell; Keene, Germany) for 2 h at 100 V. The residual binding sites on the membrane were blocked by incubation for 1 h with 5% nonfat milk in Tris-buffered saline (TBS), which was composed of 10 mM Tris-HCl, pH 7.4, and 150 mM NaCl. The membranes were then incubated for 2 h either with 1:5,000 rat monoclonal anti-galectin-3 or 1:20,000 mouse monoclonal anti-glutamine synthetase (GS; Chemicon International) for 2 h. The blots were washed three times in TBS containing 0.1% Tween-20 and probed with horseradish peroxidase-conjugated anti-rat IgG (1:2,000 dilution; Santa Cruz Biotechnology, Santa Cruz, CA) for 1 h. The membrane blots were developed using an enhanced chemiluminescence reagent kit (ECL; Amersham Pharmacia Biotech, Sweden), according to the manufacturer’s instructions.

After imaging, the membranes were stripped and reprobed using monoclonal anti-β-actin antibody as the primary antibody (Sigma-Aldrich). The optical density (OD/mm^2^) of each band was measured with a scanning laser densitometer (GS-700; Bio-Rad, Hercules, CA) and was reported as the mean±SEM. The ratios of the density of the galectin-3 band to that of the β-actin band were compared using Molecular Analyst software (Bio-Rad). The results were analyzed statistically using one-way ANOVA (ANOVA) followed by the post hoc Student–Newman–Keuls *t*-test for multiple comparisons. In all cases, a p<0.05 was considered significant.

### Immunohistochemistry

Paraffin sections (5 μm) of retinas were deparaffinized in xylene, rehydrated in a series of decreasingly diluted solutions of ethanol and pretreated with 0.01 M citrate buffer (pH 6.0) in a microwave for 3 min. After hydration, the sections were treated with 0.3% hydrogen peroxide in distilled water for 20 min to block endogenous peroxidase activity. After three washes in PBS, the sections were exposed to 10% normal goat serum (ABC Elite Kit; Vector Laboratories, Burlingame, CA) and then incubated for 1 h at room temperature (RT) with primary antibodies, including 1:500 monoclonal rat anti-galectin-3. After three washes, the sections were incubated with the secondary antibody, 1:100 biotinylated rabbit anti-rat IgG (Vector), and then avidin-biotin peroxidase complexes were formed using an Elite kit (Vector). The peroxidase reaction was developed with a diaminobenzidine substrate kit (Vector). Before sections were mounted, they were counterstained with hematoxylin. As a negative control, the primary antiserum was omitted for a few test sections in each experiment, and no specific labeling of cell bodies or fibers was detected in these sections.

For double immunofluorescence, the sections were incubated in the following order: with 10% normal goat serum for 1 h at RT, with either mouse monoclonal anti-GFAP (Sigma-Aldrich) or mouse monoclonal anti-glutamine synthetase (Chemicon) for 1 h, and then with fluorescein isothiocyanate (FITC)-labeled goat anti-mouse IgG (Sigma-Aldrich) for 1 h at RT. Then, the sections were washed and incubated with rat anti-galectin-3 overnight at 4 °C, after which the sections were washed and incubated with tetramethylrhodamine isothiocyanate (TRITC)-labeled goat anti-rat IgG (Sigma-Aldrich). To reduce or eliminate lipofuscin autofluorescence, we washed the sections in PBS. Three times for 1 h at RT, dipped them briefly in distilled H_2_O, treated them with 10 mM CuSO_4_ in ammonium acetate buffer (50 mM CH_3_COONH_4_, pH 5.0) for 20 min, then again dipped them in distilled H_2_O before returning them to PBS. The double immunofluorescence-stained specimens were examined under an FV500 laser confocal microscope (400×; Olympus, Tokyo, Japan).

## Results

### Western blot analysis of galectin-3 in pig retinas

**Figure 1:**
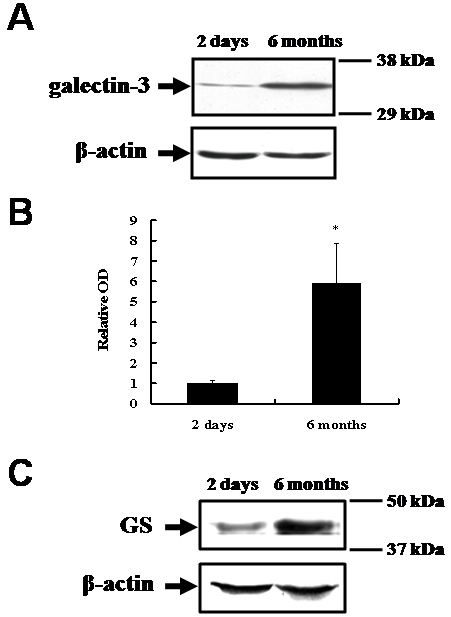
Western blot analysis of galectin-3 and glutamine synthetase in two-day-old and six-month-old pig retinas. **A**: A shows a representative western blot for galectin-3 (approximately 29–35 kDa) and β-actin (approximately 45 kDa), respectively. **B**: Graph bars (B) reveal the densitometric analysis of galectin-3 (n=3 per group). The data are presented as the mean (±SE) optical density (OD) for the level of galectin-3, relative to that of β-actin. The intensity of the galectin-3 immunoreactive band in the retinas of the six-month-old pigs was approximately six times greater than that found in the retinas of the two-day-old pigs *(p<0.05) in the description. **C**: A representative western blot for glutamine synthetase (GS; approximately 45 kDa) and β-actin (roughly 45 kDa). The level of glutamine synthetase in the six-month-old pig retina was approximately 3 times greater than that in the 2-day-old retina.

Western blot analysis was used to detect galectin-3 (molecular weight, approximately 29–35 kDa) in the homogenates of retinas from two-day-old pigs and six-month-old pigs (Figure 1). Compared with the intensity of the galectin-3 immunoreactive band in the retinas of the two-day-old pigs (relative optical density: 1.0±0.13), that in the retinas of the six-month-old pigs was significantly increased (relative optical density: 5.91±1.98; p<0.05; Figure 1A,B). This finding suggests that galectin-3 is expressed in the retina of two-day-old pigs, and its expression increases during postnatal development to the level seen in six-month-old pigs. A western blot analysis of glutamine synthetase was performed in the same samples to observe changes during postnatal development. The results indicated that the level of glutamine synthetase in the six-month-old pig retinas was significantly greater than that in the two-day-old pig retinas (Figure 1C).

### Differential localization of galectin-3 immunoreactiviy in pig retinas

**Figure 2:**
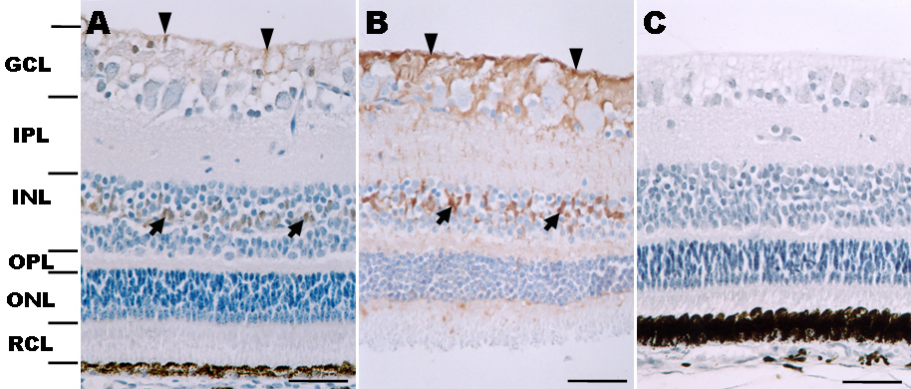
Immunohistochemical localization of galectin-3 in two-day-old and six-month-old pig retinas. **A**: In the retina of a two-day-old pig, galectin-3 immunostaining is seen in some cells in the GCL (arrowheads) and INL (arrows). Weak galectin-3 immunoreactivity was detected in the INL and outer limiting membrane. **B**: In the retina of a six-month-old pig, the pattern of galectin-3 immunoreactivity was similar to that seen in the retina of the two-day-old pig shown in (**A**). Galectin-3 was immunostained in glial cell processes from the nerve fiber layer to the outer limiting membrane. Particularly, the immunoreactivity in the GCL (arrowheads) and INL (arrows) was enhanced, compared with that in the two-day-old pig. **C**: This is a negative control (two-day-old pig retina). No specific reaction product is seen in sections incubated with nonimmune serum. Abbreviations: ganglion cell layer (GCL); inner plexiform layer (IPL); inner nuclear layer (INL); outer plexiform layer (OPL); outer nuclear layer (ONL); rod and cone layer (RCL). **A**–**C** were counterstained with hematoxylin. Scale bars represent 50 μm.

In the retinas of two-day-old piglets, galectin-3 immunostaining was observed in glial processes in the innermost nerve fiber layer and the ganglion cell layer (Figure 2A). It was also detected in cell bodies in the inner nuclear layer and in a few processes in the outer limiting membrane, while there was little staining of ganglion cells in the ganglion cell layer and neuronal cells in the inner nuclear layer and outer nuclear layer. The pattern of galectin-3 immunostaining was similar between the retinas of six-month-old pigs and two-day-old pigs, with some exceptions. In the ganglion cell layer of the six-month-old pig retinas, the galectin-3 immunoreactivity was more intense and covered a broader area (Figure 2B). In the inner nuclear layer, we observed a striking increase in the intensity of galectin-3 immunoreactivity in glial cell bodies and their processes, and across all retinal layers, including the ganglion cell layer, inner plexiform layer, outer plexiform layer, and outer nuclear layer (Figure 2B), whereas galectin-3 immunoreactivity was very weak at these sites in two-day-old pig retinas.

**Figure 3:**
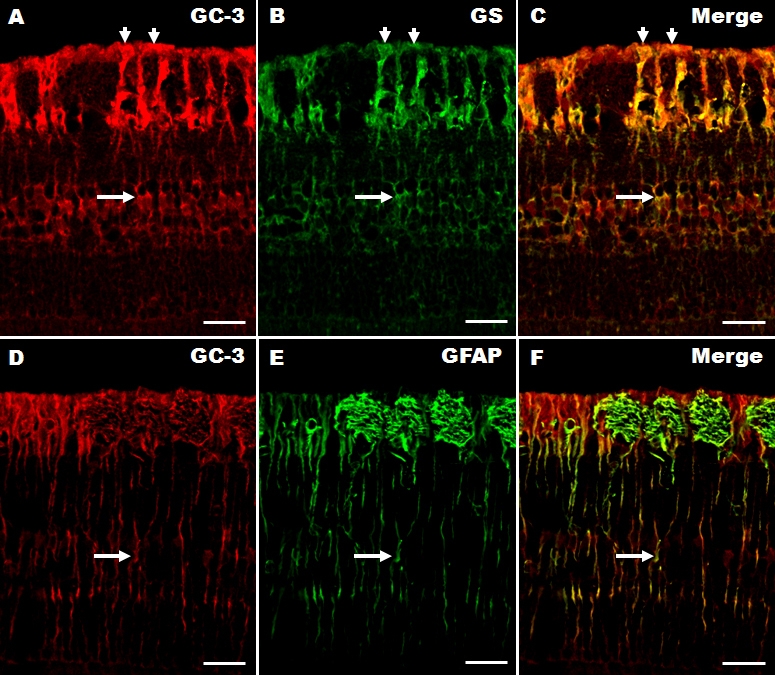
Double immunofluorescence showing colocalization of galectin-3 with either glutamine synthetase, or GFAP in six-month-old pig retinas. Galectin (GC)-3 (**A**; red fluorescence, arrows) is colocalized in most glutamine synthetase (GS)-positive cells (**B**; green fluorescence, arrows). The arrows in each figure indicate Müller cell bodies. The colocalization of galectin-3 and GS is shown in **C** (merged image). Galectin-3 (**D**; red fluorescence, arrow) was also observed in GFAP-positive cells (**E**; green fluorescence, arrow) in the retina. Colocalization is shown in **F** (merged image). The arrow in each figure (**D-F**) indicates GFAP-positive processes across all retinal layers. **C** and **F** are merged images (yellow fluorescence, arrow) of a six-month-old pig retina. Scale bars represent 50 μm.

A double-labeling experiment was performed to determine the cellular phenotype of galectin-3 expression in the pig retina. The results for six-month-old pig retinas are shown because the galectin-3 immunoreactivity was similar in both age groups. Galectin-3 was colocalized with glutamine synthetase, a marker for Müller cells [20], in the retinas of six-month-old pigs (Figure 3A-C). Most of the glutamine synthetase-positive elements overlapped with galectin-3, suggesting that Müller cells contain galectin-3 (Figure 3C, merged image). In addition, galectin-3 was also colocalized with GFAP (Figure 3D-F), suggesting that Müller cells also contain GFAP. We cannot exclude the possibility that astrocytes from the optic nerve contain galectin-3 in the innermost nerve fiber layer.

## Discussion

This is the first study to demonstrate that galectin-3 is expressed in the pig retina and that its expression increases with development. The expression of both galectin-3 and glutamine synthetase was more intense in the retinas of six-month-old pigs than in those of two-day-old pigs. This suggests that galectin-3 is detected in the retinas of postnatal pigs, and that the increased expression of galectin-3 in the adult retina is correlated with retinal development with aging. The functional roles of increased galectin-3 during retinal development are not clear. It is possible that galectin-3 plays a role in cellular protection, because the interaction of galectin-3 with a glycoconjugate on the cell surface could increase cell viability and anti-apoptotic activity [23].

A previous study has shown that the pig retina is mature one week before birth, with the exception of the photoreceptor segments, which are still differentiating [18]. This finding suggests that proliferation of pig retinal cells, including Müller cells, is complete at birth. The increased level of glutamine synthetase in the six-month-old pig may be related to the increased activity of glutamine metabolism in the retinal Müller cells during postnatal development.

Uehara et al. [12] detected galectin-3 expression in Müller cells in the rat retina after constant light exposure. Müller cells function in the clearance of extracellular glutamate via the glutamate transporter and in the synthesis of glutamine from glutamate by glutamine synthetase [22]. Given that galectin-3 on the cell surface or in secreted form has the capacity to bind carbohydrate-containing proteins, galectin-3 expressed on Müller cells may work in an autocrine manner as a receptor for secreted factors. In addition, factors secreted by retinal Müller glia exert beneficial effects on the survival of adult retinal ganglion cells and neurite regeneration in vitro, suggesting that they are effective agents for the neuroprotection of retinal ganglion cells [14]. Studies have reported that the decrease in ganglion cells in the pig retina was attributable to excessive oxidative stress [14,19], and increased galectin-3 was observed in Müller cells in the degenerative rat retina [12]. We also observed intense galectin-3 immunostaining in Müller cells throughout the six-month-old pig retinas compared to the two-day-old pig retinas. Therefore, we postulate that the increased level of galectin-3 originates from Müller cells and is directly related to the neuroprotective role of Müller cells in the course of pig retinal development. However, more studies will be needed to establish the precise role of galectin-3 in retinal development. In this study, we demonstrated that galectin-3 immunoreactivity, expressed by retinal Müller cells, was increased with postnatal development of the pigs. We postulate that galectin-3 is detected in retinal cells, especially in Müller cells, and that galectin-3 in retinal Müller cells confers cell survival in the normal aging process.
